# Oral milk exosome-PLGA nanoparticles enhance anti-tuberculosis efficacy of PBTZ169 and bedaquiline

**DOI:** 10.1016/j.isci.2026.115641

**Published:** 2026-04-08

**Authors:** Eryue Liu, Chennan Liu, Yangxue Ye, Zimo Wang, Weiyan Zhang, Lei Fu, Bin Wang, Yujin Wang, Yu Lu

**Affiliations:** 1 Beijing Key Laboratory of Drug Resistance Tuberculosis Research, Beijing Chest Hospital, Capital Medical University, Beijing Tuberculosis and Thoracic Tumor Research Institute, Beijing 101149, China

**Keywords:** biological sciences, biotechnology

## Abstract

The oral delivery of next-generation anti-tuberculosis drugs, such as PBTZ169 and bedaquiline (BDQ), is hindered by their poor solubility and low bioavailability. We, here, developed a bioinspired delivery platform that combines milk exosomes with poly(lactic-co-glycolic acid) (PLGA) nanoparticles to deliver the two drugs separately. This nanodelivery system leverages the gastrointestinal stability and mucosal penetration of exosomes, along with the high encapsulation efficiency of PLGA, significantly enhancing drug hydrophilicity and stability. In murine models, the exosome-coated nanoparticles increased plasma bioavailability by 2.5- to 4.9-fold compared with free drugs and achieved superior accumulation in target organs, while mitigating the cardiotoxicity risk associated with BDQ. This synergistic strategy, integrating synthetic and natural carriers, overcomes key pharmacological barriers, offering a promising approach to developing effective and patient-compliant oral therapies for tuberculosis and other potentially infectious diseases.

## Highlights

• ME-PLGA NPs deliver PBTZ169 or BDQ with high encapsulation efficiency
• ME-PLGA NPs enhanced oral bioavailability and achieved accumulation in lung tissues
• ME-PLGA NPs reduced bacterial load by >2.5 log_10_ in a murine TB model
• The platform mitigates BDQ cardiotoxicity and exhibits excellent biocompatibility

## Graphical abstract

**Figure:**
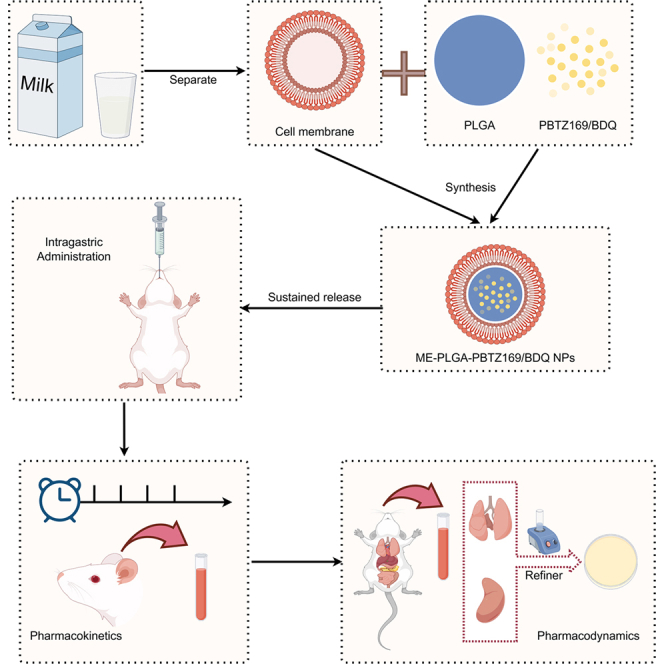

## Introduction

Tuberculosis (TB), a chronic infectious disease caused by *Mycobacterium tuberculosis* (Mtb), remains a formidable global health challenge, persistently ranking among the top ten causes of mortality worldwide. According to the World Health Organization (WHO) 2024 Global Tuberculosis Report and relevant academic studies, there were an estimated 10.8 million new TB cases globally in 2023 alone, highlighting the disease’s continuing threat to public health.^1^ While significant progress has been made in anti-TB drug development, current therapeutic regimens continue to suffer from critical limitations, including protracted treatment durations, suboptimal efficacy, and severe adverse effects, all of which contribute to poor patient compliance.^2^ These challenges necessitate the urgent development of novel treatment strategies characterized by shorter duration, reduced toxicity, and enhanced therapeutic outcomes.

Recent years have witnessed notable advances in next-generation anti-TB agents, yet several pharmacological hurdles persist. Macozinone (PBTZ169), a frontrunner in the benzothiazinone (BTZ) class of DprE1 inhibitors, demonstrates exceptional *in vitro* potency with a remarkably low minimum inhibitory concentration (MIC) of 0.2 ng/mL—significantly surpassing existing therapeutics, including bedaquiline (BDQ).^3^^,^^4^ However, its clinical translation is hindered by poor drug-like properties stemming from high lipophilicity (ClogP = 5.06), which manifests in low oral bioavailability and dose-limiting hepatotoxicity and gastrointestinal complications.^5^^,^^6^ Similarly, BDQ—while mechanistically innovative—faces substantial delivery challenges due to its extreme hydrophobicity (logP = 6.37, aqueous solubility = 0.000193 mg/mL; values from the DrugBank public property profile).^7^^,^^8^^,^^9^ These physicochemical challenges are compounded by safety considerations that require careful management in clinical settings. While BDQ has demonstrated an acceptable overall safety profile, its known corrected QT interval (QTc) interval prolongation effect necessitates monitoring during treatment, as it may increase the risk of Torsades de Pointes (TdP), a potentially life-threatening arrhythmia, particularly when co-administered with other QTc-prolonging drugs. Although hyperuricemia has been reported in some studies, it is not considered a primary or common adverse reaction associated with BDQ therapy.^10^^,^^11^^,^^12^

To circumvent these limitations, contemporary TB drug development has increasingly focused on advanced oral delivery platforms. Compared with parenteral administration, oral formulations offer superior patient compliance and clinical practicality. Given the need to overcome the physicochemical and pharmacokinetic hurdles of promising candidates like BDQ and PBTZ169 while optimizing their therapeutic safety window, the development of novel delivery systems is of significant value. Among emerging strategies, nanocarrier-based delivery systems have shown particular promise. Functionalized poly(lactic-co-glycolic acid) (PLGA) nanoparticles (PLGA NPs), for instance, have demonstrated enhanced therapeutic profiles for rifampicin and isoniazid in preclinical models, outperforming conventional formulations at equivalent doses.^13^ As an FDA-approved biomaterial, PLGA NPs provide distinct advantages, including high encapsulation efficiency, controlled biodegradability, excellent biocompatibility, and versatile surface engineering potential.^14^ Nevertheless, their clinical utility as oral delivery vehicles remains limited by premature gastrointestinal degradation and off-target biodistribution.^15^

Extracellular vesicles (EVs), particularly exosomes (30–150 nm), have recently emerged as revolutionary biological delivery vectors. Their innate phospholipid bilayer architecture, coupled with exceptional biocompatibility, prolonged circulation kinetics, and unique biological barrier-traversing capabilities, positions them as ideal drug carriers.^16^^,^^17^^,^^18^ However, intrinsic limitations in production scalability and drug loading efficiency continue to hinder their therapeutic application.^19^^,^^20^^,^^21^ In addition, milk exosomes (MEs) are highly resistant to harsh gastrointestinal tract environments.^22^^,^^23^^,^^24^^,^^25^ This has spurred innovative hybrid approaches that combine synthetic NPs with exosomal components, creating “bioinspired nanovesicles” that synergize artificial design with natural biological activity.

Building upon these research breakthroughs, our study innovatively developed an oral delivery platform that ingeniously integrates PLGA NPs with MEs to achieve separate delivery of the anti-TB drugs PBTZ169 and BDQ. This biomimetic system achieves dual optimization: (1) dramatically improving drug solubility and pharmacokinetic profiles through nanoformulation, and (2) harnessing exosomes’ innate targeting specificity and mucosal penetration capabilities for enhanced drug delivery efficiency. The incorporation of MEs further enhances the biocompatibility and physiological stability of the system. By addressing multiple pharmacological challenges simultaneously—from bioavailability enhancement to toxicity mitigation—this innovative approach represents a paradigm shift in TB therapeutics, potentially initiating a novel phase of patient-centric, high-efficiency oral treatment modalities.

## Results

### Preparation and characterization of ME-PLGA-PBTZ169 and ME-PLGA-BDQ NPs

In this study, ME-PLGA-PBTZ169/BDQ NPs were synthesized through a three-step process: (1) isolation of MEs, (2) preparation of PLGA-PBTZ169/BDQ NPs, and (3) co-incubation of MEs with PLGA-PBTZ169/BDQ NPs. The resulting NPs were lyophilized and stored at −80°C for further use.

**Figure 1:**
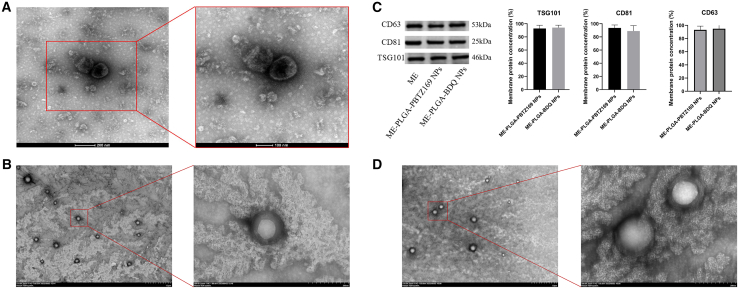
TEM images and membrane protein concentrations of ME, blank ME-PLGA NPs, and ME-PLGA-PBTZ169/BDQ NPs (A) TEM images of natural MEs and magnified views. Scale bars: 200 and 100 nm. (B and D) TEM images of ME-PLGA-PBTZ169 NPs (B) and ME-PLGA-BDQ NPs (D), with magnified views. Scale bars: 1 μm and 200 nm. (C) WB results of characteristic ME proteins and membrane protein retention rates during the preparation process (mean ± SD, *n* = 3); statistical analysis was performed using two-tailed Student’s *t* test. No significant differences were observed in the three specific proteins before and after encapsulation.

Transmission electron microscopy (TEM) imaging confirmed the characteristic cup-shaped morphology of MEs (Figure 1A). Dynamic light scattering (DLS, Table 1) analysis revealed a hydrodynamic diameter of 104.50 ± 4.86 nm for MEs, with a size distribution ranging from 30 to 150 nm, consistent with typical exosome dimensions. The relatively low polydispersity index (PDI, 0.16 ± 0.02) and high yield (BCA protein concentration: 3.29 ± 0.20 mg/mL) suggest that both the extraction method and the milk source used in this study were suitable for efficient ME isolation.

**Table 1:** Particle size, PDI, and zeta potential of ME, blank ME-PLGA NPs, and ME-PLGA-PBTZ169/BDQ NPs (Mean ± SD, *n* = 3)

Sample name	Particle size (nm)	PDI	Zeta potential (mv)
ME	104.50 ± 4.86	0.16 ± 0.02	−9.95 ± 0.12
PLGA-BDQ NPs	232.13 ± 7.24	0.15 ± 0.01	−20.44 ± 0.39
PLGA-PBTZ169 NPs	216.87 ± 7.76	0.14 ± 0.01	−20.28 ± 0.45
ME-PLGA-BDQ NPs	252.33 ± 2.91	0.15 ± 0.02	−18.26 ± 0.47
ME-PLGA-PBTZ169 NPs	252.50 ± 7.56	0.15 ± 0.02	−18.65 ± 0.83

**Figure 2:**
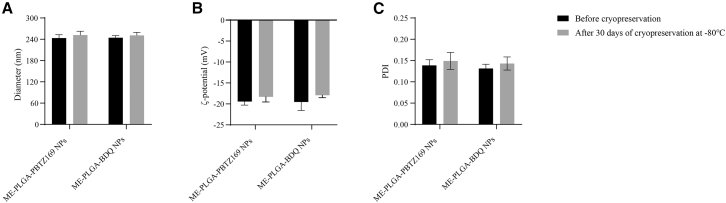
Physical stability assessment of ME-PLGA NPs after storage at −80°C for one month (A) Changes in the average particle size of nanoparticles before and 30 days after cryopreservation. (B) Changes in zeta potential before and 30 days after cryopreservation. (C) Changes in polydispersity index before and 30 days after cryopreservation. Data are presented as the mean ± SD (*n* = 3), and statistical analysis was performed using two-tailed Student’s *t* test. No statistically significant differences were observed between the groups (*p* > 0.05), indicating that the NPs remained stable after lyophilization and reconstitution under long-term low-temperature storage conditions.

The morphological characterization of ME-PLGA-PBTZ169 NPs and ME-PLGA-BDQ NPs (Figures 1B and 1C) and particle size/potential measurements (Table 1) demonstrated that the NP diameter increased correspondingly with the addition of the bilayer cell membrane coating on the NP core. Furthermore, zeta potential results indicated surface potentials of −20.28 ± 0.45 mV and −18.65 ± 0.83 mV for PLGA-PBTZ169 NPs and ME-PLGA-PBTZ169 NPs, respectively, due to the charge shielding effect from the membrane coating. Similarly, PLGA-BDQ NPs and ME-PLGA-BDQ NPs showed surface potentials of −20.44 ± 0.39 mV and −18.26 ± 0.47 mV, respectively. In this study, zeta potential measurements revealed an increase in the surface charge of PLGA after membrane coating. Compared with PLGA NPs, ME-PLGA NPs exhibited increased diameter. We evaluated the storage stability of ME-PLGA NPs at −80°C for one month. As shown in Figure 2, no significant differences were observed in the average particle size, PDI, or zeta potential of the NPs after reconstitution following lyophilization. This indicates that the NPs maintained good physical stability under long-term low-temperature storage conditions, making them suitable for subsequent experimental use. The encapsulation efficiency (EE) and drug loading capacity (DL) of ME-PLGA-PBTZ169 NPs and ME-PLGA-BDQ NPs were determined. Both drug-loaded NPs exhibited high EE (63.3% and 68.52%, respectively) and DL (15.90% and 18.13%, respectively) (Table 2). Western blot (WB) analysis confirmed the presence of exosomal marker proteins in both ME and drug-loaded ME-PLGA NPs (Figure 1). Due to dilution during the preparation process, the ME-PLGA NPs exhibited weaker band intensities in WB. However, quantitative analysis demonstrated that the procedure successfully preserved over 85% of the membrane proteins (Figure 1), confirming its efficiency. In conclusion, we successfully prepared ME-PLGA NPs.

**Table 2:** EE and DL of ME-PLGA-PBTZ169/BDQ NPs (mean ± SD, *n* = 3)

Sample name	DL (%)	EE (%)
ME-PLGA- PBTZ169 NPs	15.90 ± 1.32%	63.30 ± 5.02%
ME-PLGA- BDQ NPs	18.13 ± 2.92%	68.52 ± 7.13%

### Surface hydrophilicity of ME-PLGA NPs compared to raw APIs

The surface wettability characteristics of PBTZ169, BDQ, PLGA-PBTZ169 NPs, PLGA-BDQ NPs, ME-PLGA-PBTZ169 NPs, and ME-PLGA-BDQ NPs were evaluated via contact angle measurements after lyophilization and tablet compression. The contact angle (θ), defined as the angle formed between the tangent to the liquid-vapor interface and the solid-liquid interface at the three-phase contact point, serves as a key parameter for assessing surface wettability. θ < 90° indicates a hydrophilic surface with good liquid spreading, while θ > 90° suggests a hydrophobic surface with poor wettability.

**Figure 3:**
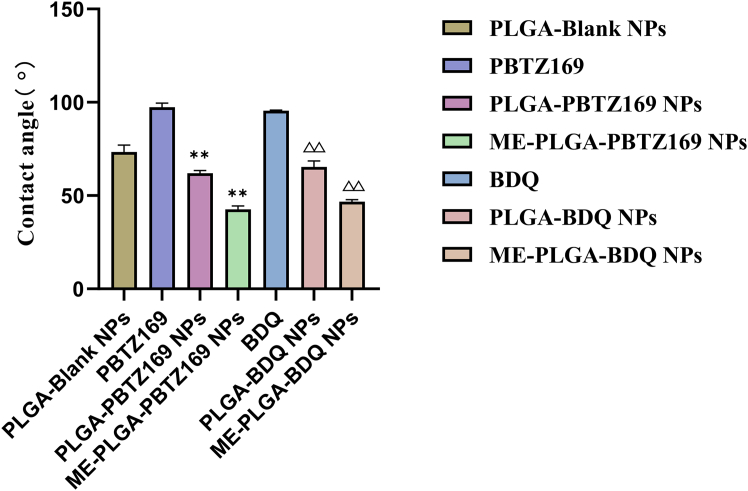
Contact angle measurements of raw drug PBTZ169, blank PLGA NPs, and PLGA-PBTZ169 NPs Data are presented as the mean ± SD (*n* = 3), and statistical analysis was performed using one-way analysis of variance (ANOVA). Compared with the PBTZ169 group, ∗∗*p* < 0.01; compared with the BDQ group, ΔΔ*p* < 0.01.

Marked differences in surface wetting behavior were observed experimentally: when water droplets were placed on the surfaces of the raw active pharmaceutical ingredient (APIs) (PBTZ169 and BDQ), the droplets exhibited a distinct spherical shape with contact angles exceeding 90°, demonstrating typical hydrophobic characteristics with minimal spreading. In contrast, on the surfaces of ME-PLGA NPs and PLGA NPs, the droplets spread rapidly, with contact angles all below 90°, which reflected the wetting behavior characteristic of hydrophilic surfaces. As shown in Figure 3, both ME-PLGA NPs and PLGA NPs displayed θ of <90°, confirmed their hydrophilic nature, whereas the raw APIs showed significantly higher contact angles (θ > 90°), indicating strong hydrophobicity.

These results demonstrate that the ME-PLGA encapsulation strategy can significantly improve surface properties of the original drug compounds, effectively enhancing their hydrophilicity and solubility.

### *In vitro* anti-mycobacterial activity of ME-PLGA-PBTZ169 and ME-PLGA-BDQ NPs

The anti-TB activity of ME-PLGA-PBTZ169 NPs and ME-PLGA-BDQ NPs was evaluated using the microplate Alamar Blue assay (MABA) against Mtb. The MICs were determined for both nanoformulations and their corresponding free drugs. Free PBTZ169 and BDQ exhibited MIC values of 0.000625 and 0.0625 μg/mL, respectively. Drug-loaded PLGA NPs and ME-PLGA NPs showed MIC values comparable to free drugs (*p* > 0.05), indicating no significant loss of anti-microbial potency after nanoencapsulation. Blank PLGA and ME-PLGA NPs (10 mg/mL) displayed no inherent anti-bacterial activity, which confirmed that the observed effects were drug dependent. Notably, while free drugs required DMSO for solubilization, the nanoformulations were readily dispersible in 0.9% saline, demonstrating significantly improved aqueous compatibility. Notably, identical MIC values were obtained for H37Ra compared to H37Rv across all tested samples (see Table S1), demonstrating equivalent susceptibility of both reference strains to the compounds and nanoformulations.

### *In vitro* cytotoxicity of ME-PLGA-PBTZ169 and ME-PLGA-BDQ NPs

**Figure 4:**
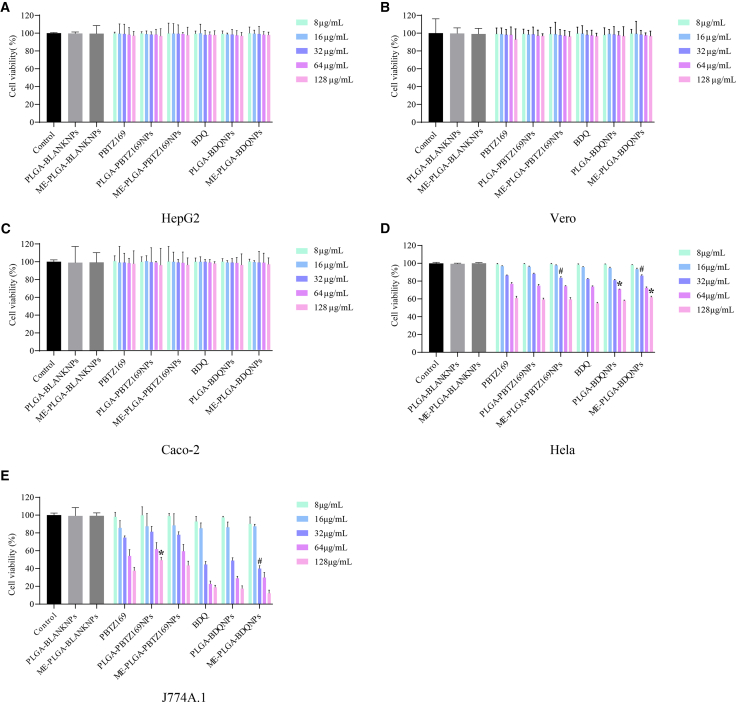
Cytotoxicity evaluation of BDQ/PBTZ169, PLGA-BDQ/PBTZ169 NPs, ME-PLGA-BDQ/PBTZ169 NPs, and two blank carrier materials in different cells (A–E) Cell viability of two blank carrier materials (10 mg/mL) and three formulations (PBTZ169 and BDQ) at various drug concentration gradients (8, 16, 32, 64, and 128 μg/mL) in HepG2 (A), Vero (B), Caco-2 (C), HeLa (D), and J774A.1 (E) cells, comparing the *in vitro* safety of different formulations at the same drug concentration. Data are presented as the mean ± SD (*n* = 3), and statistical analysis was performed using one-way analysis of variance (ANOVA). Compared with the PBTZ169 group, ∗*p* < 0.05; compared with the PLGA-BDQ NP group, #*p* < 0.05. Statistical analysis was performed for different drug formulations at the same concentration, and no significant differences were observed except for the indicated groups.

The cytotoxicity evaluation was conducted on multiple cell lines, including J774A.1, HepG2, Vero, Caco-2, and HeLa, which revealed that both ME-PLGA-PBTZ169 and ME-PLGA-BDQ NPs exhibited toxicity profiles comparable to their corresponding free drugs PBTZ169 and BDQ, respectively (see Figure 4). No statistically significant differences (*p* > 0.05) in cell viability were generally observed across all tested concentration ranges. Notably, the blank PLGA and ME-PLGA NP carriers demonstrated excellent biocompatibility in all tested cell lines, maintaining cell viability above 90%, even at an extremely high concentration of 10 mg/mL. These comprehensive findings collectively indicated that: (1) compared with free drug formulations, the nanoencapsulation process largely does not introduce additional cytotoxic effects; (2) the NP delivery system itself possesses favorable *in vitro* safety characteristics; and (3) the developed nanoformulations generally retain favorable toxicity profiles of the original drugs while offering the advantages of NP delivery.

### *In vitro* release profiles of ME-PLGA NPs in simulated gastrointestinal media

**Figure 5:**
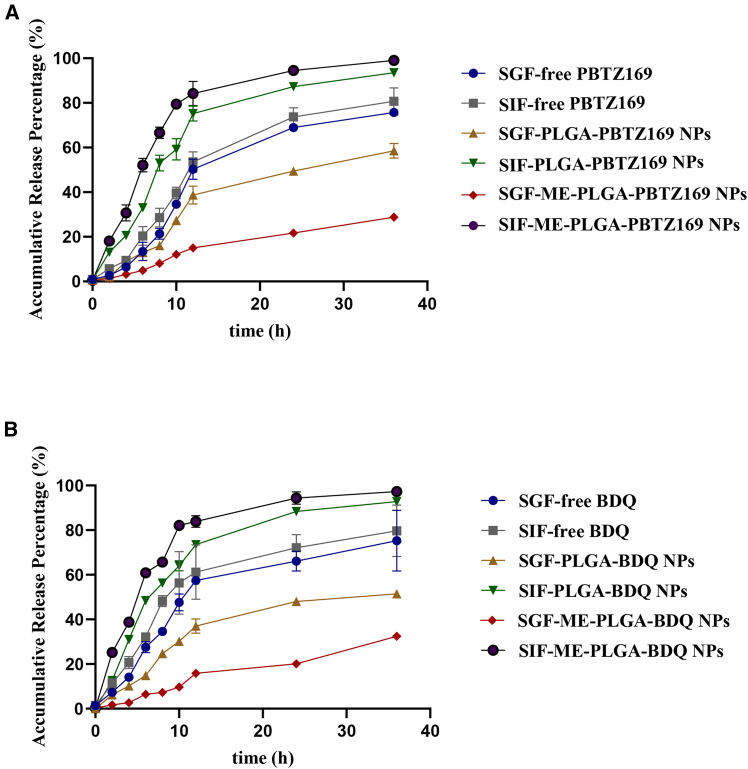
Drug release profiles of three formulations (BDQ/PBTZ169, PLGA-BDQ/PBTZ169 NPs, and ME-PLGA-BDQ/PBTZ169 NPs) under simulated gastrointestinal conditions (A) Drug release profiles of three formulations of PBTZ169 in SGF and SIF over 36 h (sampling at 0, 2, 4, 6, 8, 10, 12, 24, and 36 h). (B) Drug release profiles of three formulations of BDQ in SGF and SIF over 36 h (sampling at 0, 2, 4, 6, 8, 10, 12, 24, and 36 h). SGF, simulated gastric fluid (pH = 1.2); SIF, simulated intestinal fluid (pH = 6.8). Data are presented as the mean ± SD, *n* = 3.

The present study systematically compared the sustained-release characteristics of three drug delivery systems, demonstrating that ME-PLGA NPs exhibit superior sustained-release performance, significantly outperforming both PLGA NPs and free drugs. Analysis of the release behaviors under different pH conditions revealed that both PLGA NPs and ME-PLGA NPs showed (Figure 5) higher release rates in simulated intestinal fluid than in simulated gastric fluid, while ME-PLGA NPs displayed more stable pH-independent release properties. This ME-PLGA composite system not only maintained high stability in gastric environments but also achieved optimized release kinetics in intestinal conditions. Its unique sustained-release characteristics could effectively prolong the duration of drug action and reduce the dosing frequency, providing significant advantages for clinical therapy. Particularly in TB treatment, this delivery system demonstrated remarkable clinical application value by maintaining stable plasma drug concentrations, reducing dosing frequency, and improving patient compliance. Building on these findings, we further evaluated the system’s pharmacodynamic and pharmacokinetic profiles in TB animal models and clinical studies. These translational medical investigations will provide crucial foundations for developing more effective TB treatment regimens.

### Pharmacokinetic and bioavailability analyses of ME-coated formulations

**Figure 6:**
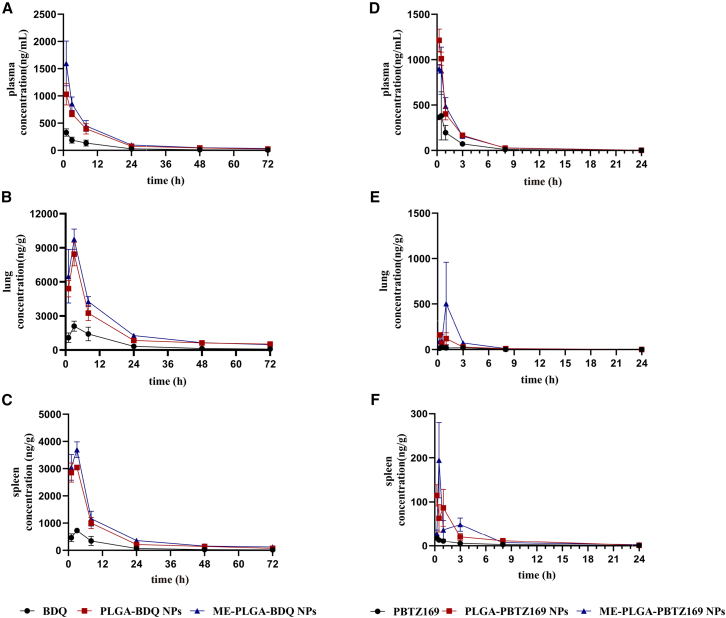
The plasma concentration-time profiles following single-dose administration of three different formulations (BDQ/PBTZ169, PLGA-BDQ/PBTZ169 NPs, and ME-PLGA-BDQ/PBTZ169 NPs) in mice (A–C) Drug concentration-time curves of BDQ in plasma (A), lung (B), and spleen (C). (D–F) Drug concentration-time curves of PBTZ169 in plasma (D), lung (E), and spleen (F). Data are presented as the mean ± SD; *n* = 3.

**Figure 7:**
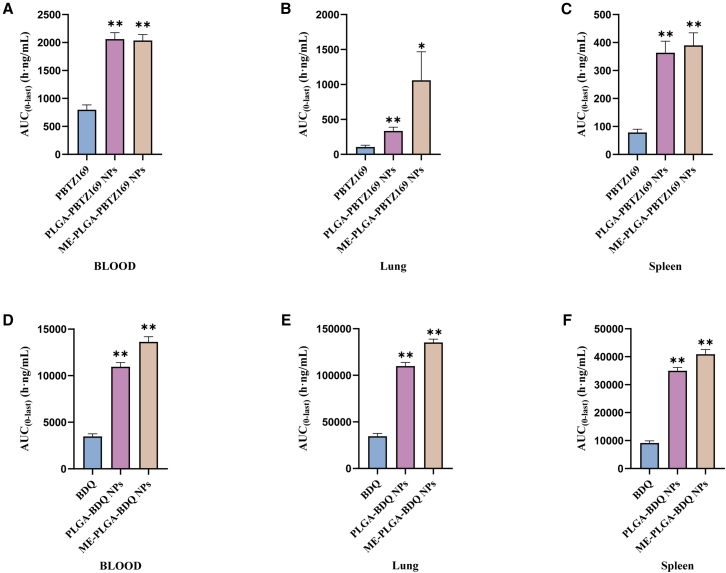
Comparative analysis of AUC values following single-dose administration of three different formulations (BDQ/PBTZ169, PLGA-BDQ/PBTZ169 NPs, and ME-PLGA-BDQ/PBTZ169 NPs) in mice (A–C) AUC values of PBTZ169 in three formulations (PBTZ169, PLGA-PBTZ169 NPs, and ME-PLGA-PBTZ169 NPs) in blood (A), lung (B), and spleen (C) after a single dose.(D–F) AUC values of BDQ in the same three formulations in blood (D), lung (E), and spleen (F). Data are presented as the mean ± SD; *n* = 3; statistical analysis was performed by one-way analysis of variance (ANOVA). Compared with the PBTZ169/BDQ group, ∗*p* < 0.05 and ∗∗*p* < 0.01.

The pharmacokinetic evaluation demonstrated that the nanoformulations, particularly the ME-coated PLGA NPs (ME-PLGA-NPs), significantly enhanced the oral bioavailability and altered the biodistribution profiles of both PBTZ169 and BDQ. Both drugs exhibited substantially increased systemic exposure after encapsulation, with notable elevations in plasma AUC and C_max_ values compared to their free drug counterparts. The terminal half-life (t_1/2_) was prolonged in plasma for both nanoformulations, indicating sustained release characteristics (Figures 6 and 7; Tables S2–S7).

Critically, the nanoformulations markedly altered the *in vivo* distribution behavior of the drugs, leading to significantly enhanced accumulation in disease-relevant tissues. Pulmonary drug concentrations were substantially elevated, particularly for ME-PLGA-PBTZ169 NPs, which achieved an approximately 10-fold increase in AUC_0–∞_ compared with the free drug. This substantial enrichment in the lungs—the primary site of TB infection—holds significant therapeutic relevance. Similarly, drug exposure in the spleen was significantly increased, which could facilitate the eradication of latent bacterial reservoirs. ME coating effectively moderated the excessively prolonged pulmonary retention observed with PLGA-BDQ NPs (reducing t_1/2_ from 71.89 h to 33.77 h), potentially reducing toxicity risks associated with drug accumulation and suggesting possibly improved biocompatibility and clearance profiles.

These results collectively demonstrated that the ME-PLGA nanoplatform successfully enhances drug solubility, achieves optimized drug release properties, and promotes favorable redistribution of drugs to therapeutically important organs. This biodistribution-shifting effect offers a promising and broadly applicable strategy for improving the therapeutic efficacy of anti-TB agents.

### *In vivo* anti-microbial efficacy of ME-PLGA-PBTZ169 and ME-PLGA-BDQ NPs

After two weeks of treatment at equivalent drug doses, a comparative evaluation of anti-bacterial activity revealed significantly enhanced therapeutic efficacy for both nanoformulations in both lung and spleen tissues.

**Figure 8:**
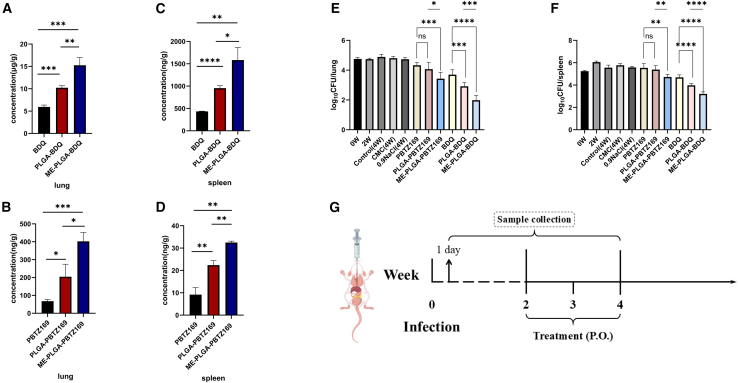
Analysis of drug concentrations *in vivo* and bacterial loads in lungs/spleens of mice after two weeks of administration of different nanoformulations (A) Drug concentrations in lung tissues of three different PBTZ169 formulations at the peak time after the last dose. (B) Drug concentrations in lung tissues of three different BDQ formulations after two weeks of administration. (C) Drug concentrations in spleen tissues of three different PBTZ169 formulations at the peak time after the last dose. (D) Drug concentrations in spleen tissues of three different BDQ formulations after two weeks of administration. (E) Bacterial colony-forming unit (CFU) counts in lung tissues after two weeks of treatment with different formulations. (F) CFU counts in spleen tissues after two weeks of treatment with different formulations. (G) Schematic timeline of infection, treatment, and organ collection in mice (experimental details are provided in STAR Methods). Data are presented as the mean ± SD (*n* = 6), and statistical analysis was performed by one-way analysis of variance (ANOVA). ∗, *p* < 0.05; ∗∗, *p* < 0.01; ∗∗∗, *p* < 0.001; ∗∗∗∗, *p* < 0.0001; NS, not statistically significant.

In lung tissue (Figure 8E), the ME-PLGA-PBTZ169 NP group demonstrated a reduction in bacterial load exceeding 1 log_10_ compared with the untreated group, performing markedly better than both the free drug group (0.4 log_10_ reduction) and the exosome-free PLGA NP group (0.6 log_10_ reduction). The ME-PLGA-BDQ NP group exhibited even stronger anti-bacterial efficacy, achieving a reduction exceeding 2.5 log_10_, which surpassed not only the free BDQ drug group (1 log_10_ reduction) but also the plain PLGA NP group (1.8 log_10_ reduction).

In spleen tissue (Figure 8F), the ME-PLGA-PBTZ169 NP group showed a 0.85 log_10_ reduction compared with the untreated group, while both the free drug and PLGA NP groups demonstrated minimal therapeutic effects. The ME-PLGA-BDQ NP group achieved a more than 2 log_10_ reduction, significantly outperforming both the free BDQ group (approximately 0.9 log_10_ reductions) and the plain PLGA NP group (approximately 1.7 log_10_ reduction).

This enhanced anti-bacterial activity correlates with our pharmacokinetic findings where both nanoformulations showed: (1) improved drug solubility in physiological conditions, (2) enhanced intestinal absorption, and (3) significantly increased bioavailability. Supporting evidence was obtained through drug concentration measurements in blood and target organs (lungs and spleen) at T_max_, which demonstrated substantially higher drug accumulation in ME-PLGA NP-treated groups (*p* < 0.05). Notably, after two weeks of treatment, both blood and tissue drug concentrations remained significantly elevated in the NP-treated groups compared with free drug administration (Figures 8A–8D), confirming the sustained-release characteristics and improved biodistribution profile of the nanoformulations. These results collectively demonstrated that the ME-PLGA platform not only enhances initial drug absorption but also maintains therapeutic drug levels over extended periods, leading to superior *in vivo* anti-bacterial outcomes.

### Effect of formulation modification on the cardiotoxicity of BDQ *in vivo*

Tg(myl7:GFP) is a transgenic zebrafish line in which GFP is expressed in cardiomyocytes, and it was used to evaluate the impact of formulation modification of BDQ on its inherent cardiotoxicity. Zebrafish larvae were treated with BDQ, PLGA-BDQ NPs, and ME-PLGA-BDQ NPs. Cardiac function, including stroke volume (SV), fractional shortening (%FS), cardiac output (CO), and heart rate (HR), was monitored through video recordings and images of zebrafish embryonic hearts.

**Figure 9:**
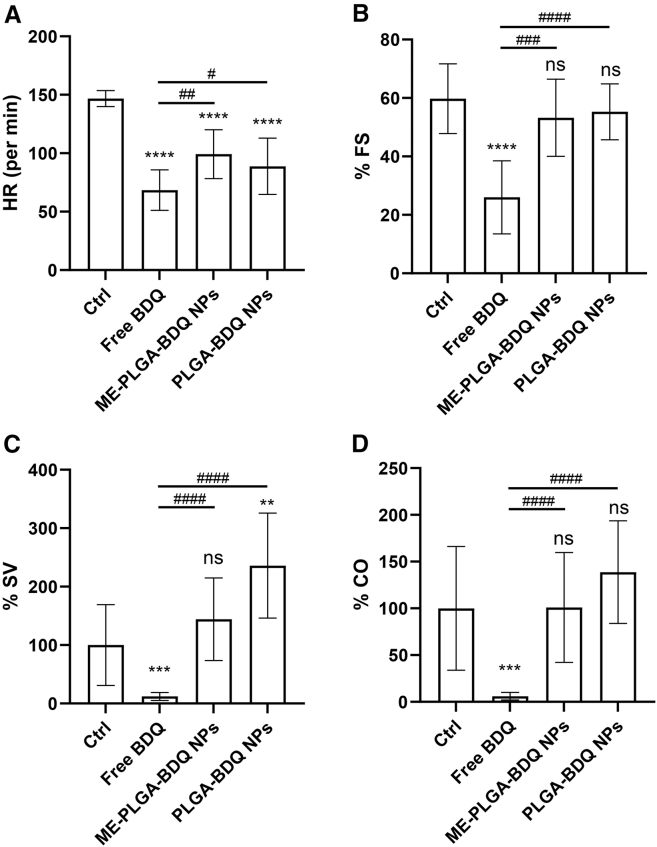
Cardiac function evaluation of zebrafish (2 dpf) exposed to free BDQ (72 μM), ME-PLGA-BDQ NPs (72 μM), and PLGA-BDQ NPs (72 μM) for 2 days (A–D) Heart rate (A), fractional shortening (B), stroke volume (C), and cardiac output (D) of zebrafish hearts. Data are presented as the mean ± SEM (*n* = 10), and statistical analysis was performed by one-way analysis of variance (ANOVA). All experiments were independently repeated three times. ∗ denotes comparison with the control group; # denotes comparison with the free BDQ group; ∗/#, *p* < 0.05; ∗∗/##, *p* < 0.01; ∗∗∗/###, *p* < 0.001; ∗∗∗∗/####, *p* < 0.0001; ns, not statistically significant.

Figure 9 shows that PLGA-BDQ NPs exhibited a certain degree of cardioprotective effect, while ME-PLGA-BDQ NPs demonstrated an optimal cardiac safety profile among all tested groups, with heart rates and cardiac function parameters closest to those of the normal control group. In contrast, free BDQ induced cardiac dysfunction, significantly reducing SV, %FS, CO, and HR compared with those in the control group. Compared with the free BDQ treatment, the formulation modifications were able to mitigate the inherent cardiovascular toxicity effects of BDQ in this zebrafish model.

## Discussion

Mtb, a recalcitrant pathogen latent in nearly a quarter of the global population, necessitates the urgent development of robust oral delivery systems for anti-TB therapeutics.^27^ While the piperazine-containing BTZ PBTZ169 (macozinone) offers significant therapeutic potential^28^^,^^29^ and BDQ remains pivotal for managing drug-resistant strains, the latter is increasingly compromised by the resistance mechanisms involving Rv0678 mutations and subsequent MmpS5L5 efflux pump overexpression.^30^ Furthermore, despite their potent anti-TB efficacy, nitro-BTZs such as BTZ043 and PBTZ169 are constrained by unfavorable physicochemical properties, specifically high lipophilicity and limited aqueous solubility.^31^ Addressing these multifaceted pharmacological hurdles, this study presents a groundbreaking biomimetic platform that synergizes synthetic PLGA NPs with natural MEs to facilitate the co-delivery of PBTZ169 and BDQ.

MEs represent a promising vector for the oral delivery of protein and peptide therapeutics, primarily attributed to their exceptional ability to traverse epithelial barriers. However, their utility is constrained by intrinsic drawbacks, including suboptimal drug loading efficiency, limited mucus penetration, and vulnerability to membrane protein loss.^20^ In contrast, biodegradable microspheres based on PLGA are widely established in medical applications due to their superior biocompatibility, low toxicity, and capacity to deliver a broad spectrum of drug molecules via multiple pathways.^32^ To synergize these complementary attributes, this study engineers a novel bioinspired drug delivery system that integrates PLGA microspheres with ME carriers, thereby addressing the individual limitations of each component while enhancing the overall therapeutic efficacy.

Key physicochemical determinants of oral drug bioavailability encompass lipophilicity, hydrophilicity, and gastrointestinal barrier permeability.^33^^,^^34^ Our characterization confirmed the successful engineering of ME-PLGA hybrid NPs optimized for oral delivery. Contact angle assays (Figure 3) revealed that ME coating substantially enhances the hydrophilicity of both PBTZ169 and BDQ, effectively mitigating the inherent constraints of these highly lipophilic agents (ClogP values: 5.06 and 6.37). This surface modification is pivotal for gastrointestinal absorption, corroborated by superior dissolution profiles in simulated intestinal fluid (Figure 5). Notably, the ME-PLGA system exhibits pH-independent release kinetics, maintaining gastric stability while enabling controlled intestinal release—a capability absent in conventional PLGA NPs.^35^ Mechanistic analysis indicated that ME-coated particles remain intact in gastric fluids but facilitate enhanced drug release in intestinal environments. This behavior likely stems from the exosomal membrane providing physical and enzymatic shielding against gastric degradation while promoting core exposure to intestinal factors such as bile salts, lipases, hydration, and endosomal acidification.^36^^,^^37^^,^^38^^,^^39^ Collectively, these factors accelerate water ingress, PLGA hydrolysis (including autocatalysis), and drug solubilization, thereby minimizing gastric loss and maximizing intestinal release compared with uncoated PLGA or free lipophilic drugs.^25^^,^^40^^,^^41^ Pharmacokinetic evaluations highlighted distinct advantages of this platform. While prior studies have established the synergistic efficacy between PBTZ169 and BDQ,^4^ our most significant finding is that ME-PLGA NPs increased the relative bioavailability of both drugs by at least 2-fold compared with free drug administration (Tables S2–S7), further amplifying therapeutic outcomes. This enhancement arises from three synergistic mechanisms: (1) exosomal membrane components facilitating mucosal penetration via intrinsic biological trafficking; (2) the PLGA core protecting against gastric degradation; and (3) the nanoformulation dramatically improving aqueous dispersibility of these hydrophobic compounds. Extended t_1/2_ confirmed the system’s sustained-release capacity. These results align with the established strategies employing PLGA NPs to boost oral bioavailability of hydrophobic drugs; for instance, previous work demonstrated a ∼4.9-fold increase in ergosterol bioavailability attributed to protective and solubilizing effects.^42^ Our study further validates nanoencapsulation as an effective strategy for delivering poorly soluble therapeutics.

From a translational standpoint, MEs offer distinct advantages in terms of scalability and cost efficiency. Industrial-scale manufacturing substantially curtails expenses, positioning MEs favorably against human- or stem cell-derived counterparts regarding production scalability, industrialization barriers, and commercialization timelines.^43^^,^^44^ These benefits stem from three primary factors: first, the abundance and low cost of raw materials, as milk—a product of large-scale agriculture—ensures stable supply at fractions of the cost associated with complex human cell cultures; second, highly optimized production workflows, where industrial techniques like tangential flow filtration (TFF) and size-exclusion chromatography facilitate continuous, automated processing of vast volumes (tens to hundreds of liters), drastically reducing extraction time and labor resources; and third, significant economies of scale, wherein unit costs decline progressively as production volume expands.

Consequently, pending successful clinical safety validation, the deployment of the ME-PLGA-BDQ/PBTZ169 nanoformulation will necessitate a multi-stakeholder collaborative framework. International procurement entities (e.g., the Global Fund) and pharmaceutical manufacturers must secure accessibility in low- and middle-income countries via tiered pricing structures. Concurrently, promoting voluntary licensing and technology transfer will enable regionalized production to guarantee long-term supply continuity. Initially positioned as an optimized regimen for drug-resistant TB, this formulation could be integrated into national strategic reserves. Thus, realizing equitable patient access demands that international organizations, industry partners, governments, and public health institutions collectively establish a sustainable translational pathway.

In pharmacodynamic assessments, ME-PLGA NPs elicited a substantial bacterial load reduction of 0.85–2.5 log units relative to free drugs (Figure 8), markedly surpassing the efficacy of uncoated PLGA counterparts. This enhanced therapeutic performance exhibited a strong correlation with observed pharmacokinetic advancements, notably a 3- to 5-fold elevation in drug accumulation within lung tissue, the primary site of TB infection. Parallel trends in splenic drug concentrations further indicate superior systemic distribution capabilities.

Several key aspects of our findings warrant emphasis. First, consistent with prior reports,^45^^,^^46^ the nanoencapsulation process preserved the intrinsic anti-microbial potency of both drugs, as evidenced by unchanged MIC values (Table 3). Second, the platform demonstrated excellent biocompatibility, confirming its suitability as an oral nanocarrier. Furthermore, ME components not only enhanced natural drug accumulation in target organs but also improved the stability of lyophilized formulations during storage, underscoring their substantial translational value. Notably, this delivery platform achieved over 3-fold higher drug concentrations in target organs at equivalent doses than free drugs, a feature that may help mitigate dose-limiting toxicities. The potential to reduce dosing frequency while maintaining therapeutic levels could significantly improve patient adherence to TB treatment regimens. Economically, for established agents like BDQ, this bioavailability-enhancing approach offers the prospect of superior therapeutic outcomes at reduced overall treatment costs, thereby alleviating the financial burden for patients who currently need expensive regimens. Moreover, compared with mammalian cell-derived exosomes, MEs offer distinct advantages in scalable production. As an abundant and accessible source, milk endows this platform with unique properties that extend its potential applications beyond TB to include gastrointestinal disorders, skin ulcers, and cancer therapeutics.^47^

**Table 3:** MICs of BDQ/PBTZ169, PLGA-BDQ/PBTZ169 NPs, ME-PLGA-BDQ/PBTZ169 NPs, and two blank carrier materials against the Mtb reference strain H37Rv *in vitro*

Sample name	MIC (μg/mL)
PLGA-blank NPs	>10
ME-PLGA-blank NPs	>10
PBTZ169	0.000625
PLGA-PBTZ169 NPs	0.000625
ME-PLGA-PBTZ169 NPs	0.000625
BDQ	0.0625
PLGA-BDQ NPs	0.0625
ME-PLGA-BDQ NPs	0.0625

In conclusion, the ME-PLGA hybrid delivery platform represents a potential paradigm shift in TB therapeutics, successfully synergizing pharmaceutical nanotechnology with biological vector engineering. By simultaneously overcoming critical barriers—specifically solubility limitations (evidenced by a ∼60% reduction in solid-liquid contact angle), bioavailability challenges (demonstrated by a 3- to 10-fold increase in target organ accumulation), and toxicity concerns—this platform paves the way for developing patient-friendly, highly efficient oral anti-TB regimens. Moreover, the versatility of this approach suggests broad applicability beyond TB, offering a promising strategy for treating other infectious diseases.

### Limitations of the study

While this study demonstrates promising improvements in drug delivery efficiency, several limitations warrant careful consideration. First, although preliminary assessments using a zebrafish model indicated that ME-PLGA-BDQ NPs exhibited the most favorable cardiac safety profile among tested groups—with heart rate and function parameters closely mirroring those of normal controls—and that uncoated PLGA-BDQ also showed some cardioprotective effects (Figure 9), these findings remain preliminary. The current evaluation lacks a systematic toxicity analysis of the modified compounds, particularly regarding long-term safety validation in advanced mammalian models. While these results suggest that the delivery system may mitigate BDQ’s inherent cardiovascular toxicity by altering release kinetics or biodistribution, definitive confirmation is pending. Furthermore, the precise mechanisms governing intestinal absorption and tissue targeting require further elucidation. Although the high lipophilicity of both drugs suggests passive diffusion as a primary transport pathway, structural modifications within our delivery system may also facilitate receptor-mediated transport processes. To address these gaps, future research should prioritize: (1) Systematic toxicity assessment: comprehensive evaluation in mammalian models to assess potential reductions in hepatotoxicity, nephrotoxicity, and cardiotoxicity. (2) Mechanistic studies: clarification of absorption pathways linked to physicochemical properties and the specific mechanisms underlying the observed reduction in cardiotoxicity. (3) Therapeutic index validation: confirmation of efficacy and safety profiles in multidrug-resistant TB (MDR-TB) models to support clinical translation. Crucially, given the well-documented correlation between BDQ’s cardiotoxicity and its high lipophilicity, it remains to be confirmed whether our delivery system effectively mitigates these adverse effects through controlled release or altered biodistribution, using comprehensive *in vivo* safety data. Consequently, during the subsequent preclinical development phase supporting an investigational new drug (IND) application, it is imperative to conduct systematic pharmacokinetic-toxicokinetic assessments—including detection of the M2 metabolite—in higher-order mammalian models (e.g., canines or non-human primates) to fully elucidate the safety profile.

## Resource availability

### Lead contact

Requests for further information, resources, and reagents should be directed to and will be fulfilled by the lead contact, Yu Lu (luyu4876@hotmail.com).

### Materials availability

This study did not generate new unique reagents.

### Data and code availability

• Data: Data reported in this paper will be shared by the lead contact upon request.
• Code: This paper does not report original code.
• Other items: Any additional information required to reanalyze the data reported in this paper are available from the lead contact upon request.

## Acknowledgments

We thank Apeng Wang from the Institute of Biotechnology, Chinese Academy of Medical Sciences, for technical assistance and providing the rotary evaporator for sample preparation. We are deeply grateful to Wenjing Liu for her expert insights on sample synthesis. These contributions do not constitute a competing interest. This work was supported by the 10.13039/501100001809National Natural Science Foundation of China (82173862) and the Beijing Municipal Administration of Hospitals’ Ascent Plan (DFL20221402).

## Author contributions

Conceptualization, E.L.; data curation, E.L.; formal analysis, E.L. and Y.L.; methodology, E.L. and C.L.; investigation, E.L., C.L., Y.Y., Z.W., W.Z., and B.W.; software, E.L.; writing – original draft, E.L.; writing – review & editing, E.L. and Y.L.; validation, L.F. and Y.W.; supervision, L.F. and Y.W.; project administration, B.W.; funding acquisition, Y.L.

## Declaration of interests

The authors declare no competing or financial interest.

## STAR★Methods

### Key resources table

**Table:** 

REAGENT or RESOURCE	SOURCE	IDENTIFIER
**Antibiotics**		

CD63	Absin	Cat# abs123229
CD81	Absin	Cat# abs126599
TSG101	bioss	Cat# bs-1365R
goat anti-rabbit IgG-HRP	Affinity	S0001, RRID:AB_2839429
goat anti-mouse IgG-HRP	Affinity	S0002, RRID:AB_2839430

**Bacterial and virus strains**		

*M. tuberculosis* H37Rv	Lab stock	ATCC 27294
*M. tuberculosis* H37Ra	Lab stock	ATCC 25177

**Cell lines**		

HepG2 cells (Male)	Lab stock originally from ATCC	ATCC HB-8065
Vero cells (Female)	Lab stock originally from ATCC	ATCC CCL-81
Caco-2 cells (Male)	Lab stock originally from ATCC	ATCC HTB-37
HeLa cells (Female)	Lab stock originally from ATCC	ATCC CCL-2
J774A.1 cells (Female)	Lab stock originally from ATCC	ATCC TIB-67

**Experimental models: Organisms/strains**		

BALB/c Mice (Female)	Beijing Vital River Laboratory Animal Technology Co., Ltd.	RRID: MGI:2161072
Zebrafish (larvae, sex ND)	China Zebrafish Resource Center(CZRC)	CZ56

**Chemicals, peptides, and recombinant proteins**		

bovine skim milk	Mengniu Dairy Co., Ltd. (Hohhot, China)	Provided commercially for this study
poly(D,l-lactide-*co*-glycolide)(50:50)-b-poly(ethylene glycol) (PLGA)	Aladdin Scientific (Shanghai, China)	34346-01-5
PBTZ169	Shanghai Hanxiang Biotechnology Co., Ltd.	20210924
BDQ	Shanghai Hanxiang Biotechnology Co., Ltd.	20210318

**Software and algorithms**		

WinNonlin® (v6.2.1)	Certara, USA	Version 6.2.1
GraphPad Prism v5	GraphPad Software, USA	Version 10.3.1

#### Animals and ethics statement

Female BALB/c mice (6–8 weeks old, specific pathogen-free) were purchased from Beijing Vital River Laboratory Animal Technology Co., Ltd. The animal study protocol was reviewed and approved by the Animal Ethics Committee of Beijing Chest Hospital, Capital Medical University (Approval No. XK2024-206, 2024). All experiments were conducted in strict accordance with the NIH Guide for the Care and Use of Laboratory Animals, and every effort was made to minimize animal suffering and the number of animals used. The study is reported in accordance with the ARRIVE guidelines.

### Experimental model and study participant details

Bacterial Strains: Mycobacterium tuberculosis H37Rv (ATCC 27294) and H37Ra (ATCC 25177) were used for *in vitro* antimicrobial susceptibility testing. Bacteria were cultured in Middlebrook 7H9 broth (supplemented with 10% OADC and 0.05% Tween 80) or on 7H10 agar plates.

Cell Lines: The following mammalian cell lines were used for cytotoxicity studies: HepG2 (human liver carcinoma), Vero (African green monkey kidney epithelial), Caco-2 (human colorectal adenocarcinoma), HeLa (human cervical adenocarcinoma), and J774A.1 (murine macrophage). Cells were cultured in DMEM supplemented with 10% fetal bovine serum (FBS) at 37°C in a 5% CO_2_ atmosphere. Cell lines were obtained from commercial sources and authenticated by the suppliers.

#### Animal models

BALB/c Mice: 72 SPF-grade female BALB/c mice, aged 6–8 weeks and weighing 18–20 g, were purchased from Beijing Vital River Laboratory Animal Technology Co., Ltd. for the *in vivo* pharmacodynamic evaluation of different formulations of PBTZ169 and BDQ. The mice were housed in an air-conditioned room designed for infected animal models, with six animals per cage. The temperature was maintained at 21 ± 2°C, humidity was kept at 55% ± 15%, and a 12-h light/dark cycle was implemented. The animals had free access to sterilized feed and filtered water. Before the experiment, the mice were allowed to acclimatize for 5 days.

An infection model was established by injecting a suspension of *Mycobacterium tuberculosis* H37Ra into the tail vein. Cultures in the logarithmic growth phase (OD600 value of 1.00–1.20) were diluted with sterile saline to an OD600 of 0.1 prior to inoculation, achieving a bacterial load of 4.0–5.0 log10 CFU in the lungs.

Treatment began 2 weeks post-infection and lasted for 2 weeks (5 days per week). The mice were randomly divided into the following groups, with 9 mice per group: (1) Active Pharmaceutical Ingredient (API) group (PBTZ169/BDQ suspended in 0.5% sodium carboxymethyl cellulose solution), (2) PLGA NPs group (PLGA-PBTZ169 NPs/PLGA-BDQ NPs dispersed in saline solution), (3) ME-PLGA NPs group (ME-PLGA-PBTZ169 NPs/ME-PLGA-BDQ NPs dispersed in saline solution), (4) Vehicle control group (0.5% sodium carboxymethyl cellulose solution and saline group), and (5) Untreated control group. The dose for all active treatment groups was 10 mg/kg (based on the mass of the active ingredient), with an equivalent dose defined as equal mass administration of the active pharmaceutical ingredient across different formulations.

6 mice from each group were euthanized on day 1 post-infection (baseline), on the day treatment began (day 0), and on the last day of treatment (day 14). Lung and spleen tissues were collected. After homogenization, tissue samples were plated onto 7H10 solid medium containing 0.4% charcoal and incubated at 37°C with 5% CO_2_ for 4 weeks for colony counting. The remaining three mice in each group were used for pharmacokinetic analysis. Lung and spleen samples were collected at the peak drug concentration time point on day 14 to measure drug concentrations.

Zebrafish: Adult Tg(myl7:GFP) zebrafish (CZRC ID: CZ56) were obtained from the China Zebrafish Resource Center and maintained under standard conditions (28°C, 14-h light/10-h dark cycle). Embryos were generated by natural crossing and cultured in E3 medium with 0.2 mM N-phenylthiourea (PTU) added from 1 day post-fertilization (dpf) to inhibit pigmentation. Larvae of mixed sexes were used for cardiotoxicity assessment at 2 dpf.

The animal study protocol was reviewed and approved by the Animal Ethics Committee of Beijing Chest Hospital, Capital Medical University (Approval No. XK2024-206, 2024). All experiments were conducted in strict accordance with the NIH Guide for the Care and Use of Laboratory Animals, and every effort was made to minimize animal suffering and the number of animals used. The study is reported in accordance with the ARRIVE guidelines.

### Method details

#### Isolation of MEs

The classical differential centrifugation (ultracentrifugation) method was used to extract MEs.^26^ To remove somatic cells and debris, pasteurized bovine skim milk was centrifuged for 30 min at 4 °C at 4000 ×g, followed by 13,000 ×g for 30 min. To precipitate milk casein, the supernatant was mixed with 0.5 M Ethylene Diamine Tetraacetic Acid (EDTA) solution (3:1, v/v) and stirred on ice for 30 min. Subsequently, the suspension underwent ultracentrifugation at a speed of 100,000 ×g (Optima TM L-100 XP, Beckman, USA) for 60 min, precipitating microvesicles and residual proteins. In the end, pellets of exosomes were obtained after centrifuging at 140,000 ×g for 90 min. The bicinchoninic acid (BCA) assay kit (Beyotime, P0010) was used to determine the exosome pellets' total protein concentration after being resuspended in 10 mM phosphate-buffered saline (PBS, pH 7.4). 20 μL of each sample or protein standard was mixed with 200 μL of BCA working solution (reagent A: regent B = 50: 1, v/v) in a 96-well plate. After incubation at 37 °C for 30 min, the absorbance was measured at 562 nm using a microplate reader. Sample protein concentrations were calculated based on the standard curve. Any ME samples with a total protein concentration below 5 mg/mL were stored at −80 °C until needed.

#### Fabrication of PLGA-PBTZ169/BDQ nanoparticles and preparation of ME-Coated nanoparticles

PBTZ169/BDQ-loaded PLGA nanoparticles (PLGA-PBTZ169/BDQ NPs) were prepared using the oil/water (O/W) single-emulsion solvent evaporation method. 10 mg of PBTZ169/BDQ and 30 mg of poly(D,l-lactide-*co*-glycolide)(50:50)-b-poly(ethylene glycol) (PLGA; Sigma-Aldrich, USA) were dissolved in 10 mL of dichloromethane (DCM; Sigma-Aldrich, USA). The PLGA-PBTZ169/BDQ NPs mixture was then added to 50 mL of 5% polyvinyl alcohol (PVA) solution (Sigma-Aldrich, USA) in distilled water, which served as the surfactant. The solution was then subjected to ultrasonication (SONICS, USA) at 40 W and 4 °C for 5 min 30 s, followed by solvent evaporation using a rotary evaporator at 25 °C. The PLGA-PBTZ169/BDQ NPs were collected by ultracentrifugation (Beckman Coulter Optima L-90 K) at 8000 ×*g* for 30 min. The pellets containing PLGA-PBTZ169/BDQ NPs were washed three times with distilled water. After lyophilization, the PLGA-PBTZ169/BDQ NPs were stored at 4°C until further use. The encapsulation efficiency (EE) and drug loading (DL) of PLGA-PBTZ169/BDQ NPs were quantified by UV-Vis spectrophotometry.

The DL and EE of PLGA-PBTZ169/BDQ NPs can be calculated using the following formulas:

$$DL\left(\%\right)=\frac{W_{\text{Drug}}}{W_{\text{All}}}\times 100\%$$

$$EE\left(\%\right)=\frac{W_{\text{Drug}}}{W_{\text{Total}}}\times 100\%$$

Where *W*_All_ represents the combined mass of the carrier and the drug, *W*_Total_ refers to the total amount of drug initially added in the experiment.

The milk-exosome was mixed with the PLGA core for cell membrane coating at a polymer-to-membrane protein weight ratio of 1:1. The mixture was then sonicated with a bath sonicator (Fisher Scientific FS30D) for 3 min. After that, excess MEs were removed by centrifugation at 12,000 ×*g* for 30 min. The pellets containing ME-PLGA-PBTZ169/BDQ NPs were washed three times with distilled water, lyophilized, and stored at −80°C until further use. To evaluate storage stability, the nanoparticles were preserved at −80 °C for 1 month, and their particle size and zeta potential were measured on the first day and the thirtieth day.

#### Physicochemical characterization of nanoparticles

The size (diameter, nm) and surface zeta potential (mV) of the nanoparticles were measured by using dynamic light scattering (DLS, NanoBrook 90plus PALS). For studying morphology, the nanoparticle samples were adsorbed onto carbon-coated copper grids (400 mesh, Electron Microscopy Sciences) and stained with 0.2 wt % uranyl acetate (Electron Microscopy Sciences). The grids were imaged on a Hitachi HT-7800 transmission electron microscope. We performed Western blot analysis to verify the integrity of isolated ME and their successful encapsulation in PLGA nanoparticles (ME-PLGA-PBTZ169 NPs and ME-PLGA-BDQ NPs). Samples were prepared using RIPA lysis buffer containing protease inhibitors. β-actin serves as a loading control. The primary antibodies used in this study include anti-CD63 (abs123229, absin), anti-CD81 (abs126599, absin), anti-TSG101 (ba-1365R, bioss). Secondary antibodies used in Western blot analysis include goat anti-rabbit IgG-HRP (S0001, Affinity) and goat anti-mouse IgG-HRP (S0002, Affinity).

#### Measurement of surface static water contact angle

The wettability characteristics of the test samples were evaluated through static contact angle measurements using a standard sessile drop technique (Model JY-82C, Chengde Dingsheng Testing Equipment). Measurements were conducted under ambient laboratory conditions (25 ± 1 °C) for: Pure drug compounds (PBTZ169/BDQ), PLGA-PBTZ169/PLGA-BDQ NPs, ME-PLGA-PBTZ169/ME-PLGA-BDQ NPs. Three replicate measurements were obtained for each sample, with results expressed as mean ± standard deviation. Before testing, all samples were equilibrated under measurement conditions for 30 min to ensure thermal stability.

#### In vitro release kinetics of drug formulations in simulated gastrointestinal media

The release profiles of PBTZ169/BDQ in three formulations (free drug, PLGA nanoparticles, and ME-PLGA nanoparticles) were assessed in simulated gastric fluid (SGF: pH = 1.2) and simulated intestinal fluid (SIF: pH = 6.8). For each formulation, 5 mg samples were suspended in 5 mL of either SGF or SIF, followed by incubation at 37 °C with continuous agitation (100 rpm) for 36 h. Aliquots were collected at 0, 2, 4, 6, 8, 10, 12, 24, and 36 h, and drug concentrations were determined by UV-vis spectroscopy at λ_max_ values of 346 nm for PBTZ169 and 333 nm for BDQ, respectively.

#### Assessment of in vitro antibacterial activity of drug-loaded formulations

The antibacterial activity of different drug formulations was evaluated by determining their minimum inhibitory concentrations (MICs) against the H37Rv and H37Ra reference strain using the Microplate Alamar Blue Assay (MABA). Five experimental groups were included: (1) free PBTZ169/BDQ, (2) PLGA-PBTZ169/BDQ NPs, (3) ME-PLGA-PBTZ169/BDQ NPs, (4) PLGA-NPs, and (5) ME group.

The assay was performed using 2-fold serial dilutions of each formulation in 96-well plates. Each well received 100 μL of bacterial suspension (2 × 10^5^ CFU/mL) in Middlebrook 7H9 broth, bringing the total volume to 200 μL. Following incubation at 37 °C for 7 days, 12.5 μL of 20% Tween 80 and 20 μL of alamarBlue reagent were added to each well. After an additional 24 h incubation, fluorescence was measured (excitation 530 nm/emission 590 nm) using a microplate reader. The MIC was defined as the lowest drug concentration that inhibited ≥90% of bacterial growth compared to drug-free controls, with *M. tuberculosis* H37Rv and H37Ra serving as the drug-susceptible control strain.

#### Evaluation of in vitro cytotoxicity

Cytotoxicity assessment of drug-loaded nanoparticle formulations (PBTZ169/BDQ, PLGA-PBTZ169/BDQ NPs, and ME-PLGA-PBTZ169/BDQ NPs) and two blank carrier materials (PLGA-NPs and ME-PLGA-NPs) performed in HepG2, Vero, Caco-2, HeLa, and J774A.1 cells using the CCK-8 assay. Cells were plated in 96-well plates at a density of 10,000 cells per well and allowed to adhere during a 24 h incubation period in DMEM at 37 °C. After the initial culture, the growth medium was replaced with fresh medium containing the test formulations at specified concentrations: blank carrier materials (10 mg/mL) or drug-loaded nanoparticle formulations (drug concentration gradient: 8, 16, 32, 64, 128 μg/mL). Cells were then incubated with these formulations for 48 h. After the 48-h treatment period, the medium in each well was carefully aspirated and replaced with 100 μL of fresh medium containing 10% CCK-8 reagent. Wells containing medium plus CCK-8 reagent without cells served as blank controls, and wells with cells in medium without any treatment served as cell-only controls (100% viability). The absorbance of each well was measured at 450 nm using a microplate reader. Cell viability was calculated as a percentage relative to the cell-only control group using the following formula:Cell Viability (%) = [(OD_sample_ -OD_blank_)/(OD_control_-OD_blank_)] ∗100%

OD_sample_ represents the OD_450_ value of wells containing cells treated with either drug-loaded formulations or blank carrier materials. OD_blank_ represents the OD_450_ values of wells containing culture medium only (blank control). OD_control_ represents the OD_450_ value of wells containing untreated cells (negative control).

#### Investigation of pharmacokinetics and bioavailability of PBTZ169 formulations in BALB/c mice

All experimental procedures involving animals were approved by the Beijing Chest Hospital, affiliated with Capital Medical University Committee on Animal Use and Care. All Mice (BALB/c, female, 6–8 weeks, 18-20g) were obtained from Beijing Vital River Laboratory Animal Technology Co., Ltd. Mice were randomly assigned to each group. The pharmacokinetic profile and relative bioavailability of different PBTZ169 formulations were evaluated following single-dose administration in BALB/c mice (*n* = 3 per group) after 12 h of fasting. Three formulations - free PBTZ169 (suspended in 0.5% carboxymethyl cellulose), PLGA-PBTZ169 nanoparticles (in normal saline), and ME-PLGA-PBTZ169 (in normal saline) - were administered orally at 10 mg/kg PBTZ169. Blood and tissue samples were collected at 0.25, 0.5, 1, 3, 8, and 24 h post-dosing. Blood samples were centrifuged within 1 h of collection to obtain plasma, which was subsequently stored at −80°C. Tissue samples (lung and spleen) were homogenized in ice-cold methanol, followed by protein precipitation with acetonitrile containing an internal standard.

The concentrations of PBTZ169 in plasma and tissue homogenates were quantified using liquid chromatography-tandem mass spectrometry (LC-MS/MS). Pharmacokinetic parameters (AUC_0-inf_, T_1/2_, C_max_, T_max_) were derived using the non-compartmental analysis module of WinNonlin (v6.2.1), with relative bioavailability determined through AUC comparisons across formulations. This comprehensive analysis demonstrated distinct pharmacokinetic behaviors and bioavailability characteristics among the different PBTZ169 delivery systems.

#### Investigation of pharmacokinetics and bioavailability of BDQ formulations in BALB/c mice

The pharmacokinetic profile and relative bioavailability of different BDQ formulations were evaluated following single-dose administration in BALB/c mice (*n* = 3 per group) after 12 h of fasting. Mice were purchased and randomly assigned to each group. Three formulations - free BDQ (suspended in 0.5% carboxymethyl cellulose), PLGA-BDQ nanoparticles (in normal saline), and ME-PLGA-BDQ (in normal saline) - were administered orally at 10 mg/kg BDQ. Blood and tissue samples collected at 1, 3, 8, 24, 48, and 72 h post-dosing were processed to obtain plasma (centrifuged within 1 h and stored at −80°C) and tissue homogenates (lung/spleen in ice-cold methanol, precipitated with acetonitrile containing internal standard). The concentrations of BDQ in plasma and tissue homogenates were quantified using LC-MS/MS. Pharmacokinetic parameters (AUC_0-inf_, T_1/2_, C_max_, T_max_) were derived using the non-compartmental analysis module of WinNonlin (v6.2.1), with relative bioavailability determined through AUC comparisons across formulations. This comprehensive analysis demonstrated distinct pharmacokinetic behaviors and bioavailability characteristics among the different BDQ delivery systems.

#### Pharmacodynamic studies

Mice were purchased and acclimatized in a Biosafety Level 2 (BSL-2) facility. A total of 100 female BALB/c mice (6–8 weeks, 18–20g) were intravenously inoculated with *Mycobacterium tuberculosis* H37Ra via the tail vein to establish an infection model. To achieve a pulmonary bacterial load of 4.0–5.0 log_10_ CFU, mid-log phase cultures (OD_600_ 1.00–1.20) were diluted to OD_600_ of 0.1 in sterile saline prior to administration. Following a 2-week infection period, treatment was initiated and maintained for 2 weeks (5 days/week) with the following groups (*n* = 9/group, mice were randomly assigned): (1) API group (PBTZ169/BDQ in 0.5% CMC solution), (2) PLGA NPs group (PLGA-PBTZ169 NPs/PLGA-BDQ NPs in normal saline), (3) ME-PLGA NPs group (ME-PLGA-PBTZ169 NPs/ME-PLGA-BDQ NPs in normal saline), (4) Vehicle controls (0.5% CMC and normal saline groups), and (5) Untreated control group. All active treatment groups were administered at a dose of 10 mg/kg (based on the mass equivalence of the active pharmaceutical ingredient). The equivalent dose is defined as the equal mass of the active drug in different formulations. We euthanized mice through CO_2_ inhalation. For sample collection and analysis, six mice per group were sacrificed at baseline (1 day post-infection, D-13), treatment initiation (Day 0, D0), and study endpoint (Day 14 post-infection, D14). Lung and spleen homogenates were plated on 0.4% (w/v) charcoal-supplemented 7H10 agar and incubated at 37°C under 5% CO_2_ for 4 weeks before CFU enumeration. The remaining three mice per group were used for pharmacokinetic analysis, with plasma, lung, and spleen samples collected at each drug’s T_max_ on D14 for drug concentration determination.

#### Zebrafish maintenance, husbandry, and cardiotoxicity evaluation

This experimental procedure was approved by the Beijing Chest Hospital affiliated Capital Medical University Committee on Animal Use and Care. Adult zebrafish were purchased from the China Zebrafish Resource Center (CZRC). The zebrafish and embryo cultures followed standard procedures. They were maintained under a 14-h light/10-h dark cycle. Embryos were generated by crossing adult zebrafish aged 2 months to 1 year. The Tg(myl7:GFP) zebrafish line (CZRC ID: CZ56) was used in this study, with individuals of mixed sexes. Embryos and larvae were cultured at 28 °C in E3 medium (5 mM NaCl, 0.17 mM KCl, 0.33 mM CaCl2, 0.33 mM MgSO4; Deepspace Bio, China). From 1 day post-fertilization (dpf) onward, 0.2 mM N-phenylthiourea (PTU; Rhawn, China) was added to prevent pigment formation and avoid interference with imaging.

Tg(myl7:GFP) zebrafish larvae were used for cardiovascular function studies. Healthy 2 dpf zebrafish larvae were employed and randomly placed into 12-well microplates, with 10–15 larvae per well, and treated with 1 mL of either free drug BDQ (72 μM) or an equivalent mass concentration of PLGA-BDQ NPs and ME-PLGA-BDQ NPs, respectively.

After a 2-day incubation period, zebrafish embryos were embedded in 1% (wt %) low-melting-point agarose to fix their orientation and position and to restrict movement. At the end of the incubation, a 15s video of the beating heart of individual larvae was recorded using a Leica fluorescence stereomicroscope M165 FC at room temperature to capture heart morphology and enable quantitative assessment of ventricular function. Ventricular function was evaluated based on heart rate (HR), stroke volume (SV), cardiac output (CO), and fractional shortening (FS).

Heart rate was determined by counting the number of heartbeats within a 15s interval. Video images were used to measure the longitudinal axis length (a) and lateral axis length (b) between myocardial borders of the ventricles at end-diastole and end-systole, respectively. Ventricular end-diastolic volume (EDV) and end-systolic volume (ESV) in larvae were calculated from heart dimensions using the formula for a prolate spheroid: V = 4/3πab^2^. SV, CO, and %FS were calculated as follows: SV = (EDV − ESV), CO = SV × HR, %FS = (Diastolic diameter − Systolic diameter)/(Systolic diameter) × 100%.

The average value of each parameter obtained from three independent biological replicates was used to assess the cardiac function of zebrafish larvae.

### Quantification and statistical analysis

CFU counts were log_10_-transformed before analysis. To compare two groups, a two-tailed Student’s *t* test was utilized, while for comparisons involving multiple groups, one-way analysis of variance (ANOVA) followed by the Newman–Keuls post hoc test was employed. All analyses were conducted using GraphPad Prism v5 (GraphPad Software, San Diego, CA), with *p* < 0.05 considered statistically significant. Here, all statistical details of the experiment can be found in the figure legends, where n represents the sample size.

### Additional resources

This study did not generate or utilize any additional resources, such as dedicated websites or databases.
