# Supplementary material for: Oral milk exosome-PLGA nanoparticles enhance anti-tuberculosis efficacy of PBTZ169 and bedaquiline
Source: iScience. 2026 Apr 8;29(5):115641. doi: 10.1016/j.isci.2026.115641 (PMC13141801; doi:10.1016/j.isci.2026.115641)
Supplement: Document S1. Figure S1 and Tables S1–S7 [file mmc1.pdf]

iScience, Volume *29*

Supplemental information

Oral milk exosome-PLGA nanoparticles enhance anti-tuberculosis efficacy of PBTZ169 and bedaquiline

Eryue Liu, Chennan Liu, Yangxue Ye, Zimo Wang, Weiyan Zhang, Lei Fu, Bin Wang, Yujin Wang, and Yu Lu

Table S1. Minimum inhibitory concentrations (MICs) of BDQ/PBTZ169, PLGA-BDQ/PBTZ169 NPs, ME-PLGA-BDQ/PBTZ169 NPs, and two blank carrier materials against Mycobacterium tuberculosis reference strain H37Ra in vitro

Sample name	MIC（μg/mL）
PLGA-Blank NPs	>10
ME-PLGA-Blank NPs	>10
PBTZ169	0.000625
PLGA-PBTZ169 NPs	0.000625
ME-PLGA-PBTZ169 NPs	0.000625
BDQ	0.0625
PLGA-BDQ NPs	0.0625
ME-PLGA-BDQ NPs	0.0625

Table S2. Pharmacokinetic parameters of PBTZ169 in plasma (Mean ± SD, n=3) following oral administration of different PBTZ169 formulations at equivalent doses in mice.

parameters(unit)	PBTZ169	PLGA-PBTZ169 NPs	ME-PLGA-PBTZ169 NPs
$t_{1/2}$ (h)	2.91	3.3	3.71
T_{max} (h)	0.25	0.25	0.25
C_{max} (ng/mL)	364.33±12.96	1212.74±71.43	898.94±27.87
$AUC_{(0\text{-}last)}$ (h·ng/mL)	798.84±86.31	2061.31±114.79	2036.15±106.82

$AUC_{(0-\infty)}$ (h·ng/mL)	802.1	2073.02	2049.34
$MRT_{(0-t)}$ (h)	2.68	2.51	2.63

Table S3. Pharmacokinetic parameters of PBTZ169 in pulmonary tissue (Mean ± SD, n=3) following oral administration of different PBTZ169 formulations at equivalent doses in mice.

parameters(unit)	PBTZ169	PLGA-PBTZ169 NPs	ME-PLGA-PBTZ169 NPs
$t_{1/2}$ (h)	1.70	2.23	3.01
T_{max} (h)	0.50	0.25	1.00
C_{max} (ng/mL)	25.82±10.03	160.28±10.78	503.25±322.61
$AUC_{(0-last)}$ (h·ng/mL)	105.97±24.93	335.39±53.22	1059.42±407.99
$AUC_{(0-\infty)}$ (h·ng/mL)	109.12	364.30	1061.44
$MRT_{(0-t)}$ (h)	2.41	1.92	2.28

Table S4. Pharmacokinetic parameters of PBTZ169 in splenic tissue (Mean ± SD, n=3) following oral administration of different PBTZ169 formulations at equivalent doses in mice.

parameters(unit)	PBTZ169	PLGA-PBTZ169 NPs	ME-PLGA-PBTZ169 NPs

$t_{1/2}$ (h)	8.29	5.11	5.60
T_{max} (h)	0.25	0.25	0.50
C_{max} (ng/mL)	17.68±0.38	114.84±14.04	194.65±60.56
$AUC_{(0\text{-}last)}$ (h·ng/mL)	78.49±11.61	363.71±41.26	389.96±44.63
$AUC_{(0\text{-}\infty)}$ (h·ng/mL)	89.14	372.71	410.13
$MRT_{(0\text{-}t)}$ (h)	6.14	4.27	4.27

Table S5. Pharmacokinetic parameters of BDQ in plasma (Mean ± SD, n=3) following oral administration of different BDQ formulations at equivalent doses in mice.

parameters(unit)	BDQ	PLGA-BDQ NPs	ME-PLGA-BDQ NPs
$t_{1/2}$ (h)	25.32	27.94	30.74
T_{max} (h)	1	1	1
C_{max} (ng/mL)	327.62±30.01	1031.02±79.89	1596.50±167.62
$AUC_{(0\text{-}last)}$ (h·ng/mL)	3486.49±268.20	10954.28±451.79	13632.88±549.28
$AUC_{(0\text{-}\infty)}$ (h·ng/mL)	3759.08	11897.16	15114.88
$MRT_{(0\text{-}t)}$ (h)	13.34	13.76	13.25

Table S6. Pharmacokinetic parameters of BDQ in pulmonary tissue (Mean ± SD, n=3) following oral administration of different BDQ formulations at equivalent doses in mice.

parameters(unit)	BDQ	PLGA-BDQ NPs	ME-PLGA-BDQ NPs

$t_{1/2}$ (h)	25.18	71.89	33.77
T_{max} (h)	3	3	3
C_{max} (ng/mL)	2106.12±180.90	8441.94±421.38	9757.96±364.33
$AUC_{(0\text{-}last)}$ (h·ng/mL)	34511.28±3008.85	109903.39±3908.65	135298.71±3453.99
$AUC_{(0\text{-}\infty)}$ (h·ng/mL)	37668.21	164732.92	158472.7
$MRT_{(0\text{-}t)}$ (h)	23.02	70.31	31.74

Table S7. Pharmacokinetic parameters of BDQ in spleen (Mean ± SD, n=3) following oral administration of different BDQ formulations at equivalent doses in mice.

parameters(unit)	BDQ	PLGA-BDQ NPs	ME-PLGA-BDQ NPs
$t_{1/2}$ (h)	28.09	30.98	30.51
T_{max} (h)	3	3	3
C_{max} (ng/mL)	727.66±37.31	3183.78±83.34	3561.40±190.65
$AUC_{(0\text{-}last)}$ (h·ng/mL)	9128.62±774.27	34984.04±1153.27	40905.96±1636.16
$AUC_{(0\text{-}\infty)}$ (h·ng/mL)	9970.51	38513.71	46010.27
$MRT_{(0\text{-}t)}$ (h)	13.09	14.24	13.35

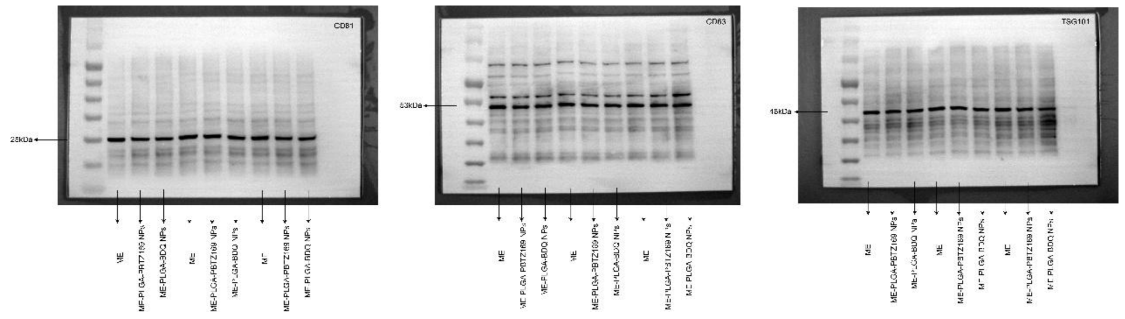

Figure S1. Uncropped Western Blots
